# The Impact of PD Check-In, a Model for Supported Self-Managed Maintenance of Speech on the Quality of Life of People with Parkinson’s Disease: A Phase 1 Study

**DOI:** 10.3390/brainsci12040433

**Published:** 2022-03-24

**Authors:** Ann Finnimore, Deborah Theodoros, Anna Rumbach

**Affiliations:** 1 School of Health and Rehabilitation Sciences, The University of Queensland, Brisbane, QLD 4067, Australia; d.theodoros@uq.edu.au (D.T.); a.rumbach@uq.edu.au (A.R.); 2 Speech Pathology Department, The Prince Charles Hospital, Metro North Hospital & Health Service, Brisbane, QLD 4032, Australia

**Keywords:** Parkinson’s Disease, speech, self-management, quality of life

## Abstract

Quality of life (QoL) for people with Parkinson’s Disease (PD) is diminished by speech and communication changes. The impact of PD Check-In, an intervention for supported self-managed maintenance of speech following LSVT LOUD^®^, on QoL of people with PD was investigated. Sixteen people with PD and dysarthria completed LSVT LOUD followed by PD Check-Ins up until 24 months post-treatment. Self-rated QoL and voice handicap scales were used to determine the psychosocial and perceived impact of PD Check-In on the speech and voice of people with PD. The perceived impact of PD Check-In on speech and voice was also sought from 15 communication partners (CPs). A significant treatment effect for time was identified for the Dysarthria Impact Profile (DIP), Voice Handicap Index (VHI), and Voice Handicap Index-Partner (VHI-P) (*p* < 0.05). There was no significant effect for time for the Parkinson’s Disease Questionnaire (PDQ-39). Planned comparisons of timepoints for DIP, VHI, and VHI-P showed no significant differences (*p* > 0.01). Comparison of perceived voice handicap by people with PD and CPs revealed no significant differences (*p* > 0.01). The impact of PD Check-In on QoL of people with PD and CPs for 24 months post-LSVT-LOUD is unclear. Self-reported outcome measures alone do not fully capture changes in QoL in PD.

## 1. Introduction

Speech and communication changes associated with Parkinson’s Disease (PD) contribute to a diminished quality of life (QoL) for those diagnosed with the progressive neurological condition, and their families and caregivers [1,2]. The emergence of prevalent symptoms in speech and communication occurs independently of the severity of symptoms [3] from pre-diagnosis across the span of the condition [1,4,5]. Up to 90% of people diagnosed with PD experience hypokinetic dysarthria [6,7], a speech impairment with hallmark features that include hypophonia, hoarse vocal quality, monoloudness, monopitch, articulatory distortion, and variable rate [8]. A further impact of PD on communication may present in cognitive–linguistic deficits in the processing and production of language and reading comprehension [9,10], and physical and emotional changes, including depression, fatigue, and fluctuations due to medication cycle [1,2,11].

People with PD and their close communication partners (CPs) have described the considerable impact of deficits in speech and communication on their QoL. Changes in self-perception, such as feeling inadequate, less independent, self-conscious, less talkative, and unsure of relationships and dynamics of social interactions are experienced by people with PD in everyday communication [1,12]. Emotional responses include anxiety, frustration, depression, and fear [13]. Altered social participation, culminating in withdrawal from everyday living activities, social networks, and vocational roles due to the challenges in accessing, initiating, and sustaining social interaction contributes to diminished QoL for people with PD [2,11,13,14]. Researchers have sought further understanding of the impact of speech and communication changes for people with PD with the addition of the perspectives of CPs. Studies that compared people with PD and CP perceptions of speech-related QoL of people with PD reported no significant difference in perceptions between groups [3,15,16,17]. Though the studies found agreement between people with PD and CPs’ perception of QoL for people with PD, varying trends in participant perceptions were described. Although families and caregivers noticed increased self-consciousness and decreased social interaction in people with PD, they were not able to understand the full impact of the speech and communication changes associated with PD on the person [16]. Parveen and Goberman [17] described a trend where CPs perceived the impact of voice and speech change more positively than self-ratings by people with PD [17]. Previous studies of the impact of speech and communication changes for people with PD suggest comparisons of perspectives of people with PD and CPs have the potential to provide insight into the impact of QoL and inform speech–language pathology (SLP) intervention for both groups [3,15,16,17].

Despite the well-documented efficacy of the LSVT LOUD intensive speech treatment for PD [18,19], long-term maintenance of the speech gains remains equivocal, with some studies showing maintenance in vocal intensity at two years post-treatment [20], and others reporting less convincing evidence [21,22,23]. The positive impact of treatment and maintenance of speech over time on the QoL of people with PD has not been substantiated. At two years post-LSVT-LOUD, Wight and Miller [21], based on self-reported responses of seven participants using the Voice Handicap Index (VHI) [24], found no significant QoL effect compared with pre-treatment. Loud and Proud, a face-to-face group therapy intervention [22], reported no statistically significant change in QoL from pre- to post-group intervention on the Communicative Effectiveness Index (CETI) [25] and the Quality of Communication Life Scale (ASHA QCL) [26]. Group therapy via telerehabilitation, eLOUD and Proud [23], found no significant QoL effect comparing pre-, post-, and three months post-group intervention intervals using the Dysarthria Impact Profile (DIP) [27] and The Parkinson’s Disease Questionnaire (PDQ-39) [28]. 

The negative psychosocial impact of persistent speech and communication symptoms after SLP intervention, however, has been reported by people with PD in several studies. In a study by Spurgeon et al. [29], participants reported frustration and disappointment when the initial benefits of speech treatment were not maintained, with feelings of embarrassment and anxiety ongoing in daily communication [29]. Experiences and perspectives of people with PD following SLP intervention were elicited in semi-structured interviews with 24 people with PD by Yorkston et al. [2]. Participants reported persistent physical and cognitive investment in speaking, and feelings of boredom and embarrassment with repetitive practice drills, highlighting the need for SLP intervention and ongoing support to be inclusive of the unique social contexts of communication for individuals with PD [2]. Similarly, people with PD have expressed their need for regular and ongoing SLP access, not only to maintain function, but also to support the constant psychosocial impact of speech and communication on QoL [13].

To this end, PD Check-In, a novel intervention for SLP-supported self-managed maintenance of speech and communication for people with PD following LSVT LOUD, was developed and trialed in a clinical service for ambulatory rehabilitation. The development of PD Check-In and the underpinning framework has been described by Finnimore, Theodoros, and Rumbach [30]. This model involves a broadened role for SLPs to support people with PD to self-evaluate their functional ability in terms of social participation, and to employ strategies for speech and communication in defined intervals of self-management. PD Check-In provides a balance of clinical feedback and SLP-guided semi-structured discussion based on partnership with people with PD and family and close CPs; self-reflection on communication and quality of everyday life; identification of facilitators and barriers for maintenance of speech and communication; modelling and rehearsal of LSVT LOUD skill; motivational goal-setting and shared development of strategies for goal attainment.

This study aimed to investigate the impact of PD Check-In on the QoL for people with PD over twenty-four months following LSVT LOUD. Specifically, the study aimed to: (1) determine the self-reported psychosocial impact of speech and communication changes for people with PD; (2) determine the perceived impact of voice and speech changes on the QoL of people with PD from the perspective of CPs; and (3) compare the perceived impact of voice and speech changes of CPs and people with PD over time. It was hypothesized that a SLP-supported self-managed maintenance program following LSVT LOUD would improve QoL of people with PD. Additionally, it was hypothesized that the perceived impact of voice and speech changes for people with PD would align with those of their CPs.

## 2. Materials and Methods

### 2.1. Research Design

A small group repeated measures Phase 1 study design was selected for this preliminary investigation in a clinical setting [31].

### 2.2. Participants

Recruitment of two cohorts of participants was conducted as a sample of convenience in an ambulatory rehabilitation setting. Twenty people with hypokinetic dysarthria due to PD, and 19 close CPs, met eligibility criteria for the study. All participants were 18 years or older, proficient in English, and independent in providing informed consent. All participants with PD had a confirmed diagnosis of idiopathic PD with dysarthria provided by a neurologist or medical practitioner with expertise in PD. People with a history of neurosurgical management of PD, including Deep Brain Stimulation (DBS), remained eligible to participate. Exclusion criteria were applied to potential participants with PD with severe cognitive impairment, including diagnosed dementia and co-existent severe medical, neurological, and psychiatric conditions. Further prerequisites for inclusion of people with PD related to suitability for LSVT LOUD, which was established prior to recruitment to the study in an initial SLP assessment conducted by a clinician independent of the research team. Additionally, confirmation of vocal symptoms consistent with PD, and suitability of people with PD for intensive voice treatment were obtained by an assessment performed by an otolaryngologist, independent of the study. From the PD cohort, there was 20% (n = 4) attrition of participants from the study, leaving a data set of 16 participants with PD. Reasons for withdrawal related to revision of neurological diagnosis (n = 2), seeking additional SLP services (n = 1), and relocation inter-state (n = 1). Nine men and seven women with a mean age of 70.68 years (SD = 8.53, range = 48–82 years) comprised the PD cohort. On average, PD participants were 5.9 years post-diagnosis (SD = 4.59, range = 0.6–18 years). The median stage of PD progression was 2.5, with a range from Stage 1–4 [32]. Two people who had undergone DBS participated in the study. Participants with PD received a perceptual rating of severity of dysarthria in the initial comprehensive SLP assessment. Severity ratings ranged from mild to severe dysarthria, with 12 participants with mild dysarthria, two rated as mild-to-moderate, one as moderate, and one as severe (see Table 1). PD Check-In, as defined by objective speech outcomes, has been evaluated using the same PD cohort. These results will be published in a separate report, and more information can be found there [33].

Close CPs, unfunded for their companionship, were nominated by participants with PD. CPs with communication impairment, including cognitive and hearing deficits, were not eligible for inclusion. The recruited cohort of CPs comprised 19 participants, as one participant with PD did not identify a close CP. The complete data set of 16 CPs (11 females and 4 males) remained after attrition of three participants secondary to the withdrawal of three participants with PD from the study, and the withdrawal of a fourth CP due to frailty (see Table 1).

### 2.3. Procedure

Participants with PD received the LSVT LOUD program followed by PD Check-In, a maintenance intervention, delivered at 6 and 12 weeks, and 6, 12, and 24 months post-LSVT-LOUD. Each individual face-to-face PD Check-In session, delivered by the principal investigator, lasted approximately one hour. CPs were invited to attend PD Check-In sessions at the discretion of the participants with PD. LSVT LOUD was delivered in accordance with protocol by an accredited SLP [34]. Pre- and post-LSVT-LOUD evaluations were administered approximately one week either side of the intensive treatment. In keeping with the conduct of this study within a clinical service, the principal investigator delivered LSVT LOUD to participants, and engaged SLPs independent to the study for clinical evaluation at pre-and post-treatment timepoints. Scheduling of LSVT LOUD and PD Check-In appointments was responsive to participant convenience and clinical availability. Participants were required to complete the LSVT LOUD daily home program [35], and to attend the five PD Check-In intervention sessions.

A typical PD Check-In session comprised the clinical evaluation of vocal intensity of phonation and speech and fundamental frequency range of the person with PD, followed by a semi-structured discussion between the SLP and the person with PD, possibly accompanied by their CP, targeting the development of self-management principles for long-term speech maintenance. Clinical outcomes formed the basis of collaborative reflection on the maintenance of vocal intensity using comparative data from pre- and post-LSVT-LOUD. Clinical issues identified by the SLP regarding voice production received therapeutic advice and intervention. The semi-structured discussion, based on a topic guide [30], explored the perceptions of the person with PD regarding their self-management and functional maintenance of speech in daily life. The semi-structured format of discussion enabled the SLP to support the development of self-evaluation through probing for success and failure, and facilitators and barriers in communication. Collaborative goal setting and the identification of strategies for attainment concluded the PD Check-In intervention, and supported effective self-management for the period of maintenance ahead. Self-rated QoL and perceived speech and voice impact scales were completed by participants with PD and CPs at timepoints in the treatment and maintenance phases of the study. For participants with PD, ratings occurred pre- and post-LSVT-LOUD, and at PD Check-In sessions at 12 weeks, and 6, 12, and 24 months post-treatment. The perspectives of CPs were sought pre- and post-LSVT-LOUD, and at 12 and 24 months post-treatment (see Table 2).

### 2.4. Outcome Measures

Paper-based standardized self-rating scales were provided to participants with PD and CPs, at varying timepoints from pre-LSVT-LOUD to 24 months post-treatment (see Table 2), measuring (a) the impact of PD and changes in speech and communication on everyday life for people with PD (i.e., Dysarthria Impact Profile (DIP) [27] and Parkinson’s Disease Quality of Life Questionnaire (PDQ-39) [28]) and (b) changes in voice and speech as perceived by the participant (i.e., Voice Handicap Index (VHI) [24]) and their CPs (the Voice Handicap Index-Partner (VHI-P) [36]). Participants with PD who required physical assistance in completing forms sought support from their CPs or an SLP independent of the research team. If a communication partner was unable to attend a session when the VHI-P was due for completion, forms were supplied for completion at home as close as possible to the PD Check-In date, and returned via post.

#### 2.4.1. Dysarthria Impact Profile

The DIP measures the psychosocial impact of dysarthria from the perspective of the speaker. The questionnaire comprises 48 questions presented in five sections: (A) the effect of dysarthria on me as a person; (B) accepting my dysarthria; (C) how I feel others react to my speech; (D) how dysarthria affects my communication with others; and (E) dysarthria relative to other worries and concerns [27]. Statements were worded positively or negatively. Participants rated each statement on a five-point scale weighted either positively (1 = strongly agree, 3 = unsure, 5 = strongly disagree) or negatively (1 = strongly disagree, 3 = unsure, 5 = strongly agree). In Section E, participants nominated four aspects of PD, in addition to speech changes, and placed the five symptoms in order of concern (1 = most concern, 5 = least concern). The total score of the DIP comprised the sub-scores from Section A through E. The total score and sub-scores from the DIP were used in the data analyses. For the person with PD, a lower score on the DIP represents an increased negative impact of dysarthria on QoL, and a higher score indicates a lessening of impact [27].

#### 2.4.2. PDQ-39

PDQ-39 explores the impact of PD on QoL through a standardized self-rated questionnaire across eight health and lifestyle domains. People with PD are required to respond based on their experiences in the period one month prior to completing the questionnaire. The domains are presented with a variable number of: mobility (10), activities of daily living (6), emotional wellbeing (6), stigma (4), social support (3), cognitive impairment (4), communication (3), and bodily discomfort (3). Participants were required to independently rate their response with a mark for each statement using a 5-point ordinal scale (0 = never, 1 = occasionally, 2 = sometimes, 3 = often, 4 = always or unable to do at all). The total score for each domain was calculated by dividing the sum of the participant scores by the potential score for the domain multiplied by 100. A Summary Index Score was calculated as the sum of the total scores in each domain. A lower score on PDQ-39 represents a better QoL for the person with PD [28]. For the purposes of this study, only the communication component of PDQ-39 was analyzed.

#### 2.4.3. VHI

The VHI is a questionnaire seeking the speaker’s perception of the handicap they experience due to their disordered voice [24]. The 30-statement index probes the domains of emotional (E), functional (F), and physical (P) aspects of living with a voice disorder, with ten statements in each domain. Participants were required to provide independent responses to rate their experience in terms of frequency of occurrence for each statement, placing a mark on a five-point ordinal scale from 0–4 (never = 0, almost = 1, never = 2, sometimes = 3, almost always = 4, always = 5). Additionally, participants were required to rate their perception of the presence or severity of their voice disorder at the time of the evaluation using a four-point scale (normal, mild, moderate, severe). The combined scores of each domain resulted in a total score out of 30. A higher score on the VHI represents an increase in the perception of voice handicap, and a lower score represents a lesser perception of handicap. The sub-scores for domains and the total score were included in the data analyses for the study.

#### 2.4.4. VHI-P

CPs provided their perceptions of the handicap experienced by people with PD by completing the VHI-P [35] at 12 and 24 months post-treatment. Derived from the VHI [24], the VHI-P comprises 30 statements which seek the communication partner’s perspective on the emotional (E), functional (F), and physical (P) impact of a disordered voice on the person with PD. CPs rated how frequently they felt the person with PD experienced each of the ten statements in the domains on a 5-point ordinal scale from 0–4 (never = 0, almost = 1, never = 2, sometimes = 3, almost always = 4, always = 5). CPs were also required to rate the presence or severity of a disordered voice for the person with PD at the time of the evaluation, using a four-point scale (normal, mild, moderate, severe). The summed scores of each domain resulted in a total score out of 30. A higher score on the VHI-P represents an increase in the CP perception of voice handicap for the person with PD, and a lower score represents a lesser perception of handicap. The sub-scores for individual domains and the total score were included in the data analyses.

### 2.5. Data Analysis

Data analyses were performed using SPSS (Version 26; IBM, Armonk, NY, USA). Non-parametric procedures were performed for the ordinal data from each measure. Data from the DIP, PDQ-39, VHI, and VHI-P were analyzed using the Friedman Test with an alpha level of 0.05 to determine whether PD Check-In had a significant effect on QoL over time. Where significance was found, planned comparisons using Wilcoxon signed-rank tests were conducted at pre-to-post, post-to-12-m, and post-to-24-m. A stringent alpha level of *p* < 0.01 was used to account for multiplicity of testing [37]. Data from VHI and VHI-P were analyzed using the Mann–Whitney test with an alpha level of *p* < 0.01 to account for multiplicity of testing to determine if a significant difference in perceived voice and speech handicap for people with PD existed between participants with PD and CPs across time points.

## 3. Results

**Figure 1:**
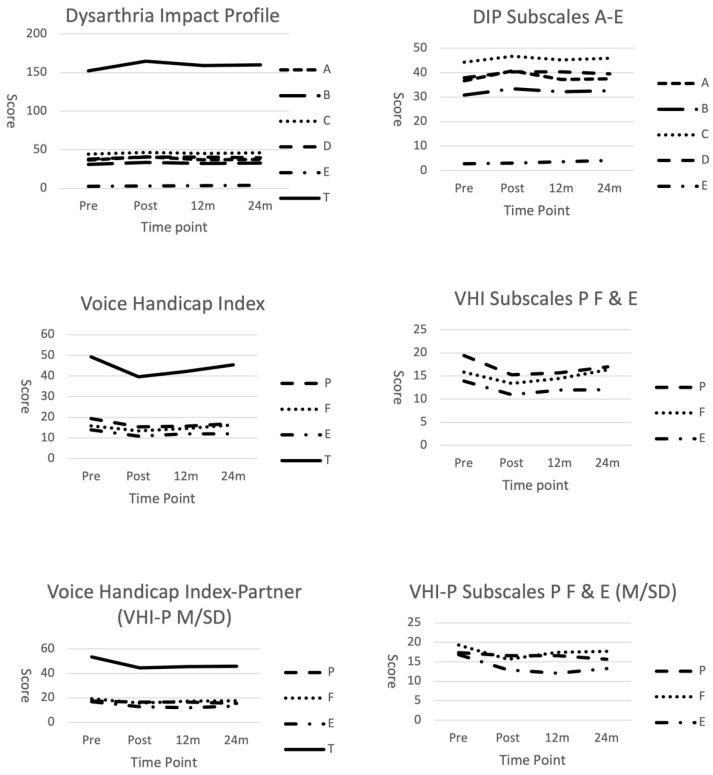
Mean total scores and subscale scores of Dysarthria Impact Profile, Voice Handicap Index, and Voice Handicap Index-Partner. Pre = pre-LSVT-LOUD; Post = post-LSVT-LOUD; 12 m = 12 months post-LSVT-LOUD; 24 m = 24 months post-LSVT-LOUD. Dysarthria Impact Profile [27]: higher score represents lessening of psychosocial impact. Voice Handicap Index [24]: lower score represents lessened perception of voice handicap. Voice Handicap Index-P [35]: lower score represents partners’ lessened perception of voice handicap.

Results of the Friedman’s tests revealed a significant effect for time for the DIP (x^2^ = 319.12; *p* = 0.0001), VHI (x^2^ = 141.77; *p* = 0.0001), and VHI-P (x^2^ = 136.92; *p* = 0.0001). No significant effect for time was identified for the PDQ-39 (x^2^ = 0.625; *p* = 0.987). Planned comparisons of the DIP, VHI, and VHI-P data using the Wilcoxon signed-rank test and an alpha level of *p* < 0.01 failed to identify any significant differences across the time points for these outcome measures (see Table 3). However, a series of line graphs provide a descriptive view of the trends in the data across the subscales and total scores for these outcome measures. For the DIP, the total score and subscale data reflect a slight lessening of the impact of the speech disorder on the cohort post-treatment, which is maintained above the pre-treatment level across time (See Figure 1). For the VHI and VHI-P, there is a trend of a decrease in the overall voice handicap immediately post-treatment, with maintenance of this effect below pre-treatment levels across 24 months. The physical, functional, and emotional subscales of these measures reflect a similar pattern (See Figure 1).

There were no significant differences between participants with PD and CPs regarding the perception of voice handicap across the physical, functional, and emotional domains, and total scores across time points (see Table 4).

## 4. Discussion

This study aimed to determine the perceived impact of PD Check-In on the QoL of people with PD. Although the outcome measures used in this study failed to reflect significant changes in QoL as a result of PD Check-In, trends in the data suggest that some improvement and maintenance of QoL over time for people with PD may have occurred.

The long-term QoL gains reported in this and previous studies in the maintenance of speech and communication after treatment were not statistically supportive of the need expressed by people with PD for a sustained impact of SLP intervention on communicative QoL [2,29]. Wight and Miller [21] reported no significant difference in the total scores of VHI for participants at 24 months post-LSVT-LOUD treatment, although analysis of VHI subscales showed statistically significant gain in the functional domain at 24 months post-treatment. Similarly, neither face-to-face group therapy [22] nor group therapy delivered via telerehabilitation [23] following LSVT LOUD demonstrated statistically significant differences in QoL for people with PD.

Participants in the current study previously reported a high level of satisfaction with PD Check-In. Qualitative statements indicative of QoL change were received in the absence of a significant maintenance of vocal intensity (SPL) of conversational monologue 24 months following LSVT LOUD [30]. Changes in vocal intensity (SPL) have been shown to not predict communication effectiveness [3] and QoL outcomes [38]. Though there are numerous studies reporting different models for speech maintenance post-treatment, our ability to compare outcomes for QoL across studies is limited by the use of different self-reported QoL measures. Minimally-demonstrated change in QoL for people with PD, using self-reported measures, highlights the limitations of such measures to adequately capture change. Additionally, self-reported measures do not adequately identify factors beyond clinical outcomes in voice and speech, which lead to change in QoL for people with PD and CPs.

Though the DIP, PDQ-39, and VHI have been used extensively as validated patient reported outcomes in studies in PD, speech, and communication [39,40,41], measures of QoL in PD have been found to not fully encompass the impact of speech and communication changes on the lives of people with PD, and their families and caregivers [42]. QoL measures have been shown to not align with self-perception, variable coping strategies, and individual reactions of people with PD [12,16,43]. The value of employing effective QoL measures in SLP practice is to ensure assessment and intervention are inclusive of the psychosocial influences on everyday communication [12,37], and to evaluate the impact of intervention on the maintenance or improvement of communicative quality of life [44]. Without a true indication of the long-term impact of PD Check-In on the QoL of people with PD, our contribution to sustained outcomes is inconclusive.

The DIP, PDQ-39, VHI, and VHI-P shared limitations in the extent to which they fully represented the impact of speech and communication changes on QoL for people with PD. The DIP extensively probed the impact of features of dysarthria on QoL without specificity for PD [27]. The completion of the 48-item scale required physical and cognitive endurance from participants. The inclusion of complex linguistic constructions, for example, double negatives (“I do not get angry when I cannot make myself understood”) and items which were presented in positively- and negatively-weighted paired statements (“Even when I am not speaking I feel that I am a different person now/My speech difficulty has not changed me fundamentally as a person”) [27], were challenging for some participants, and resulted in contradictory responses [32]. Atkinson-Clement and colleagues [40], in confirming the psychometric properties of the DIP in French, reworded potentially ambiguous statements, and reduced the 5-point response scale to a binary choice, “agree/disagree”, to facilitate the independent completion of the scale by people with PD [40]. Effortful completion of the questionnaire highlighted the potential for the loss of meaningful information in self-reported QoL outcomes [45] due to cognitive–linguistic challenges faced by many people with PD [9].

The limitations of PDQ-39 for this study were largely related to the breadth of the scale [28]. In order to adequately represent the changes in communicative QoL [37], self-reporting by participants with PD was confined to communication items only at post-LSVT-LOUD, and then at 12 weeks, and 6 and 12 months post-treatment (see Table 2). The sharpened focus on the communication items to the exclusion of other domains did not facilitate further analysis and insight into QoL changes associated with speech and communication for people with PD. Difficulties in extracting meaningful information from broad measures with few items dedicated to speech and communication has fueled investigations into more specific QoL measures for people with PD and others experiencing diminished communicative participation [37,46]. People with PD, when responding to PDQ-39 items, are instructed to confine their reflections on their function and PD experience to the period one month prior to completing the questionnaire. Fluctuations in perceived QoL in the month preceding PD Check-In may positively or negatively influence the self-evaluation of a longer period of communicative QoL between PD Check-In time points.

For people with PD, self-perception of the clinical features of articulation, and voice quality may be less developed than their awareness of overall changes in communication, and loss of speech clarity in functional contexts [12,16]. Although frequently selected as a patient-reported outcome for PD [45], the specificity of the VHI to the single characteristic of voice, albeit a hallmark, may distract or limit people with PD from the broader perception of speech and communication in daily life [1,12]. The final component of the VHI, self-rated voice quality as normal, mildly, moderately, or severely impaired, can be challenging for some participants who perceive persistent dysphonia as their “normal” voice quality, thus limiting the reflection of change in the perceived impact of voice over time [12].

The perspective of CPs on the impact of voice and speech changes on the QoL of people with PD over time was investigated. Though changes in CPs’ perception across time points on VHI-P were not significant, there was a trend of lessened perceived impact of speech in all domains from pre- to post-LSVT-LOUD. Importantly, at 24 months post-treatment, the perceived lessened impact of speech on quality of life for participants with PD remained above the pre-treatment level in all domains. This trend in CPs’ perception is suggestive of the importance of the maintenance of voice and speech following treatment for QoL for people with PD and CPs.

A further aim of this study was to compare the perceptions of CPs with those of people with PD regarding the impact of voice and speech changes on QoL for people with PD over time. Previous studies which compared self-reported perceptions of voice handicap reported CPs generally perceived less impact of speech changes on the QoL of people with PD; however, no statistically significant differences were found [16,17]. Though the current study found no significant differences in perceived voice and speech handicap between PD and CP cohorts, further comparison with the previous studies is limited, as neither study investigated the QoL impact over time. Miller and colleagues [16] compared the perceptions of people with PD with those of their CPs before and after diagnosis of PD using a semantic differential questionnaire comprising 22 bipolar adjective pairs [16], whereas Parveen and Goberman [17] compared the QoL impact of speech and motor-related changes in PD at a single time point.

The influence of PD Check-In on the perceived impact of speech on the QoL is not clear; however, the descriptive data presented in Figure 1 indicates a positive trend of lessened perceived handicap, which was maintained for 24 months following LSVT LOUD. Though the difference in perspectives of people with PD and CPs in this and previous studies is not significant, there is a need to consider the clinical implications of supporting the needs and expectations of people with PD and CPs for communicative QoL [3,15,16,17]. The clinical importance in seeking the perspectives of both groups is to determine if differences in perception exist within the communication partnership [3]. Working in partnership with people with PD and CPs is an important element of the self-managed maintenance framework which underpins PD Check-In [30]. Similarities and differences in perceptions of people with PD and CPs have the potential to influence partnership in speech maintenance. Where differences occur between people with PD and CPs, intervention has the opportunity to support the alignment of perspectives for mutually-agreed goals for speech maintenance, and to collaboratively develop strategies for success in daily communication. Early identification of differences in the views of people with PD and CPs facilitates timely provision of education to support the communication relationship [47].

## 5. Limitations and Future Directions

Elements of the design and conduct of the study may have limited the capturing of the QoL impact of PD Check-In for people with PD and CPs. As a Phase 1 study [31], a small cohort of participants was investigated with no matched control participants. The absence of significant differences in self-reported QoL and perception of speech and voice-related QoL over time may be attributed, in part, to the small sample size. Heterogeneity in the PD cohort, recruited to the study as a sample of convenience, may also have contributed to the inconclusive results. As a study conducted within clinical practice, the participant cohort reflected the heterogeneous nature of PD in severity and duration of symptoms, and treatment. Though the variation in PD characteristics may have influenced the outcome, the relevance of clinical studies in PD which reflect the heterogeneous presentation of people with this condition to SLP services warrants consideration. Self-reported QoL outcomes were interpreted with consideration of the size and nature of the data sample. The absence of matched controls limited the interpretation of the impact of PD Check-In on QoL and extrapolation of these results to broader PD and SLP contexts.

The paper-based questionnaires were completed by participants in the latter part of a PD Check-In session, following the clinical outcome measures and semi-structured discussion. The number of questionnaires presented varied according to the time point post-LSVT-LOUD (see Table 2); however, they were delivered when participants were possibly experiencing isolated or combined effects of physical, cognitive, and emotional fatigue [1,2,9,11].

The design of the questionnaires and the paper-based format proved to be functionally challenging for some participants due to writing and visual difficulties associated with PD [43,45] resulting in slow, effortful written responses. Infrequent assistance from CPs as scribes for participants with PD may have introduced some bias in responses [43]. Negative attitudes to completing questionnaires previously expressed by people with PD and CPs [32] may have influenced participants’ engagement with self-reporting QoL outcomes.

Limitations of individual measures have been discussed. In the time since this longitudinal study commenced, considerable advances have been made in the validation of tools which enable SLPs to more effectively assess the psychosocial influences on speech and communication, and to evaluate the impact of intervention on QoL [37,41,45]. The inclusion of scales specific to communicative QoL, delivered in a variety of media to increase access for people with PDPD, and to reduce the burden on respondents [45], is a strong recommendation for future investigations. Expansion of the SLP role to be more inclusive of communication context, and to evaluate the psychosocial impact of intervention, aligns well with the focus on self-evaluation of social participation in daily life in the PD Check-In semi-structured discussion [30]. Though the impact of PD Check-In on QoL was difficult to quantify, an investigation into the factors associated with life satisfaction [48] found general self-efficacy, a developmental focus in PD Check-In, to have a significant association with life satisfaction. With this in mind, the next step in this investigation, involving qualitative analysis of the semi-structured discussions in the intervention, may provide more insight into the impact of PD Check-In for people with PD and CPs.

## 6. Conclusions

The impact of PD Check-In on the QoL of people with PD and their CPs over 24 months following LSVT LOUD was inconclusive. Though sustained, if not measurably improved, QoL through supported maintenance of speech and communication following treatment is the primary goal for this intervention, the evaluation of QoL is compounded by the individuality of the experience of PD, and the unique factors which determine QoL for individuals. The development of measures which capture the individual communication and social participation experiences of people with PD, and accommodate the physical, emotional, and cognitive barriers faced by them and their CPs, is necessary for engagement with self-reported QoL measures. PD Check-In offers long term SLP connection for people with PD and CPs following treatment, employing a balance of clinical and psychosocial communication goals for a better life with PD. Qualitative analysis of the semi-structured discussion component of PD Check-In may enhance our understanding of QoL for people with PD and their CPs, and contribute to a refinement of evaluation measures which encompass the impact of speech and communication intervention in PD. Future studies in the form of randomized controlled trials are required to further investigate the efficacy of PD Check-in as a model for speech maintenance and QoL for people with PD and their families and CPs.

## Figures and Tables

**Table 1:** Demographics of participants with PD.

Participant	Age (Years)	Gender	Time Post PD Diagnosis (Years)	Hoehn and Yahr Stage	Severity of Dysarthria	DBS	Relationship of Communication Partner
1	65	Male	5.0	2.0	Mild	N	Spouse
2	70	Male	10.0	3.0	Mild	N	Spouse
3	71	Female	0.8	2.0	Mild	Y 18 mths post-Tx	Spouse
4	70	Male	0.6	2.0	Mild-mod	N	Spouse
5	74	Male	5.0	3.0	Mild	N	Spouse
6	75	Female	2.0	3.0	Mild	N	Spouse
7	70	Female	3.0	3.0	Mild	N	Daughter-in-law
8	77	Male	3.0	1.0	Mild	N	Spouse
9	61	Male	11.0	1.0	Mild	N	Spouse
10	82	Female	8.0	1.0	Mild	N	Spouse *
11	79	Male	6.0	4.0	Mild	N	Spouse
12	81	Male	7.0	3.0	Mild	N	Spouse
13	73	Female	5.0	2.0	Mild	N	Spouse
14	62	Male	18.0	3.0	Severe	Y 6 years pre-Tx	Spouse
15	74	Female	9.0	2.0	Mild-mod	N	Daughter
16	48	Female	1.0	3.0	Moderate	N	Spouse

DBS = Deep Brain Stimulation; Tx = treatment; * communication partner withdrew prior to 24 months post-LSVT-LOUD.

**Table 2:** Quality of life rating scale schedule.

		Treatment		Maintenance				
		Pre-LSVT-LOUD	Post-LSVT-LOUD	6 Weeks	12 Weeks	6 Months	12 Months	24 Months
PWPD	DIP	X	X				X	X
	PDQ-39	X All	X Comm		X Comm	X Comm	X Comm	X All
	VHI	X	X				X	X
CP	VHI-P	X	X				X	X

PWPD = Person with Parkinson’s Disease; CP = Communication Partner; DIP = Dysarthria Impact Profile [27]; PDQ-39 = Parkinson’s Disease Questionnaire-39 [28]; VHI = Voice Handicap Index [24]; VHI-P = Voice Handicap Index-Partner [35]. PDQ-39: All = all domains; Comm = communication domain only; 6 weeks, 12 weeks, 6 months, 12 months, 24 months = time post-LSVT-LOUD.

**Table 3:** Analysis of planned comparisons for DIP, VHI, and VHI-P from pre-to-24 months post-LSVT-LOUD.

	Pre Median (IQR)	Post Median (IQR)	Z	*p* *	Post Median (IQR)	12 m Median (IQR)	Z	*p* *	Post Median (IQR)	24 m Median (IQR)	Z	*p* *
DIP												
A.	39.00	40.50	−1.924	0.054	40.50	39.00	−1.665	0.096	40.50	38.50	−1.892	0.058
	(12.00)	(9.75)			(12.00)	(7.75)			(7.75)	(12.00)		
B.	32.00	34.50	−1.147	0.251	34.50	32.00	−0.570	0.568	34.50	33.00	−1.038	0.299
	(4.50)	(10.50)			(10.50)	(6.50)			(10.50)	(6.50)		
C.	44.50	46.50	−1.340	0.180	46.50	45.00	−0.692	0.489	46.50	46.50	−0.171	0.864
	(8.00)	(6.25)			(11.25)	(8.00)			(8.00)	(8.00)		
D.	38.50	41.00	−1.110	0.267	41.00	41.00	−0.288	0.820	41.00	41.00	−0.127	0.899
	(8.00)	(10.00)			(8.25)	(8.00)			(8.00)	(7.25)		
E.	2.50	3.00	−0.998	0.318	3.00	4.00	−1.392	0.164	3.00	4.00	−2.271	0.023
	(2.75)	(1.00)			(2.75)	(2.75)			(2.75)	(2.75)		
Total	153.50	165.0	−2.558	0.011	165.00	159.50	−0.540	0.589	165.00	161.00	−0.884	0.377
	(33.00)	(19.75)			(33.00)	(20.00)			(34.25)	(33.00)		
VHI												
P	21.50	16.00	−1.734	0.083	16.00	16.00	−0.835	0.404	16.00	17.00	−1.383	0.167
	(9.25)	(8.00)			(9.25)	(7.50)			(19.75)	(9.25)		
F	16.50	10.50	−0.598	0.550	10.50	11.50	−1.663	0.096	10.50	14.00	−2.181	0.029
	(9.75)	(9.75)			(9.75)	(8.75)			(14.5)	(9.75)		
E	13.50	7.50	−1.039	0.299	7.50	9.50	−1.170	0.242	7.50	10.50	−0.906	0.365
	(15.25)	(13.00)			(13.00)	(10.75)			(13.00)	(13.25)		
Total	53.50	35.50	−1.475	0.140	35.50	37.00	−1.061	0.289	35.50	42.00	−1.591	0.112
	(31.00)	(25.00)			(31.00)	(28.50)			(46.00)	(31.00)		
VHI-P												
P	20.00	17.00	−0.525	0.599	17.00	18.00	−0.317	0.751	17.00	17.00	−0.629	0.529
	(6.00)	(10.00)			(6.00)	(9.00)			(9.00)	(6.00)		
F	21.00	15.00	−1.849	0.064	15.00	18.00	−1.652	0.099	15.00	18.00	−1.262	0.207
	(9.00)	(10.00)			(9.00)	(10.00)			(20.00)	(9.00)		
E	19.00	15.00	−2.029	0.043	15.00	12.00	−0.824	0.400	15.00	14.00	−1.056	0.291
	(5.00)	(7.00)			(5.00)	(12.00)			(21.00)	(5.00)		
Total	58.00	46.00	−1.819	0.069	46.00	51.00	−0.856	0.392	46.00	48.00	−0.398	0.691
	(25.00)	(27.00)			(25.00)	(30.00)			(45.00)	(25.00)		

* significance at *p* < 0.01. Pre = pre-LSVT-LOUD; Post = post-LSVT-LOUD; 12 m = 12 months post-LSVT-LOUD; 24 m = 24 months post-LSVT-LOUD. DIP = Dysarthria Impact Profile [27]: A = effect on people with PD; B = acceptance; C = reactions of others; D = communication with others; E = dysarthria relative to other concerns. Higher score represents lessening of psychosocial impact. VHI = Voice Handicap Index [24]: P = physical; F = functional; E = emotional. Lower score represents lessened perception of voice handicap. VHI-P = Voice Handicap Index-Partner [35]: P = physical; F = functional; E = emotional. Lower score represents partners’ lessened perception of voice handicap.

**Table 4:** Analysis of comparison of VHI and VHI-P across domains from pre-to-24 months post-LSVT-LOUD.

	VHI Median; (IQR)	VHI-P Median; (IQR)	Z	*p*
P1	21.50; (19.75)	20.00; (9.00)	−0.752	0.470
P2	16.00; (9.25)	17.00; (6.00)	−0.357	0.740
P3	16.00; (8.00)	18.00; (10.00)	−0.418	0.682
P4	17.00; (7.50)	17.00; (9.00)	−0.635	0.545
F1	16.50; (14.50)	21.00; (20.00)	−1.050	0.299
F2	10.50; (9.75)	15.00; (9.00)	−1.486	0.140
F3	11.50; (9.75)	18.00; (10.00)	−1.446	0.151
F4	14.00; (8.75)	18.00; (10.00)	−0.674	0.520
E1	13.50; (15.25)	19.00; (21.00)	−1.108	0.281
E2	7.50; (13.00)	15.00; (5.00)	−1.230	0.232
E3	9.50; (13.25)	12.00; (7.00)	−0.495	0.626
E4	10.50; (10.75)	14.00; (12.00)	−0.832	0.423
T1	53.50; (46.00)	58.00; (45.00)	−0.534	0.599
T2	35.50; (31.00)	46.00; (25.00)	−1.029	0.318
T3	37.00; (25.00)	51.00; (27.00)	−0.870	0.401
T4	42.00; (28.50)	48.00; (30.00)	−2.97	0.770

VHI = Voice Handicap Index [24]: lower score represents lessened perception of voice handicap. VHI-P = Voice Handicap Index-Partner [35]: lower score represents partners’ lessened perception of voice handicap. IQR = interquartile range. P = physical domain; F = functional domain; E = emotional domain; T = total score. 1 = pre-LSVT-LOUD; 2 = post-LSVT-LOUD; 3 = 12 months post-LSVT-LOUD; 4 = 24 months post-LSVT-LOUD.

## Data Availability

Participant data, although not publicly available, may be provided in response to requests that are within the Research Committees’ approvals.
